# Comparative effectiveness and cost-effectiveness of policies for provisioning rabies post-exposure vaccines

**DOI:** 10.1016/j.vaccine.2025.128178

**Published:** 2026-02-27

**Authors:** Joel Changalucha, Elaine Ferguson, Martha M. Luka, Kennedy Lushasi, Eleanor Rees, Danni Anderson, Husna Hoffu, Samweli Gwakisa, Z. Mtema, Maganga Sambo, Kimera Sharadhuri, Lwitiko Sikana, Athumani Lupindu, Jonathan Yoder, Felix Lankester, Katie Hampson

**Affiliations:** a Department of Veterinary Medicine and Public Health, Sokoine University of Agriculture, Tanzania; b Department of Environmental Health and Ecological Science, Ifakara Health Institute, Ifakara, Tanzania; c Boyd Orr Centre for Population and Ecosystem Health, School of Biodiversity, One Health and Veterinary Medicine, University of Glasgow, UK; d Ministry of Health, Tanzania; e Paul G. Allen School for Global Health, Washington State University, USA; f Global Animal Health Tanzania, Tanzania

**Keywords:** One health, Lyssavirus, Gavi, Integrated bite case management, IBCM, Dog-mediated rabies, Zero by 30

## Abstract

**Introduction:**

The burden of rabies remains high in low-income countries, where limited access to life-saving post-exposure prophylaxis (PEP) leads to preventable deaths. Lack of evidence on the implications of PEP provisioning strategies impedes policy development. We evaluate three PEP strategies under consideration in Tanzania, comparing current limited access, improved access with free provision, and free provision combined with Integrated Bite Case Management (IBCM).

**Methods:**

We examined data from IBCM implementation on PEP delivery practices, healthcare-seeking, and rabies risk across four Tanzanian regions. Using these data within a decision tree model, we evaluate health outcomes and economic impacts of proposed PEP policies from the healthcare provider perspective, projecting vaccine requirements, costs, and deaths across Tanzania over a five-year period (2026–2030).

**Results:**

We project approximately 59,000 (95 % PI: 32,000–96,000) rabies exposures nationwide over five years. With this exposure risk, current PEP access leads to around 800 deaths annually (95 % PI: 400–1200). Improved PEP access increases patients starting PEP, driving vaccine requirements to around 24,000 (95 % CI: 14,000–34,000) vials per year and reducing deaths by >40 %. Introducing IBCM reduces total projected deaths over 2026–2030 from 2300 to 2000 while remaining highly cost-effective at $181 per death averted.

**Conclusion:**

Tanzania's current PEP practice fails to meet the demand for rabies prevention, leading to preventable deaths. Free PEP is a more effective alternative, and implementing IBCM would further strengthen the country's rabies response and accelerate progress toward the target to end human rabies deaths by 2030.

## Highlights

• Each year, we estimate that around 800 people die of rabies annually in Tanzania.
• Our decision tree model is developed to inform rabies PEP policy recommendations.
• Current PEP is predicted to prevent 6000 deaths in Tanzania from 2026-2030; wider access could avert ∼2000 more
• We recommend Integrated Bite Case Management to strengthen PEP provision, improve surveillance and monitor impacts.

## 1 Introduction

Human rabies continues to be a serious public health concern in low- and middle-income countries in Africa and Asia, where it causes an estimated 59,000 deaths annually [1,2]. Beyond these health costs, human rabies imposes a significant burden on the wider economy and livelihoods of affected families and communities. Timely post-exposure prophylaxis (PEP) given to rabies-exposed individuals prevents the disease's fatal onset and is a cost-effective intervention that can alleviate the disease burden [2]. Mass dog vaccination is critical to control the source of rabies, but where dog rabies continues to circulate before elimination is reached, PEP remains as a vital measure. PEP includes immediate wound washing, administration of the rabies vaccine, and, for severe exposures, the application of rabies immunoglobulins (RIG) [2]. Investing in PEP can rapidly save lives, reduce healthcare costs, prevent loss of productivity, and minimise the economic strain on communities and healthcare systems in endemic areas. Understanding the full benefits of rabies prevention through PEP is essential for developing effective public health policies and for guiding resource allocation.

Since the initial successful rabies post-exposure vaccinations administered by Louis Pasteur over 100 years ago [3], the rabies vaccine has been extensively used to prevent human rabies, and various post-exposure protocols have been developed. The original Pasteur protocol used a nerve tissue vaccine, which has since been replaced by modern cell culture vaccines administered intramuscularly (IM) or intradermally (ID). The Essen and Zagreb post-exposure vaccination regimens require five and four IM doses, respectively, and were the earliest regimens used with cell culture vaccines [4,5]. One IM dose requires an entire vaccine vial, irrespective of whether the vial is 1 mL or 0.5 mL in size. In contrast, ID regimens require fractional doses; for example, the Updated Thai Red Cross (UTRC) regimen requires four doses of two ID injections of 0.1 mL each, administered over one month [[6], [7], [8]]. Since each vial contains multiple doses, ID regimens allow for vial sharing among multiple patients. Consequently, if patients attend clinics on the same day, fewer vials are required to complete the regimen. Evidence has demonstrated that both IM and ID regimens can be safely abridged. The latest WHO-recommended regimens are the updated Essen IM, which requires four vials, and the ID 1-week regimen, which requires three fractional doses (2 × 0.1 mL injections for each, totalling 0.6 mL) given in one week [2]. Table 1 details these post-exposure vaccination regimens.

**Table 1:** Summary of PEP regimens implemented and recommended for use in Tanzania. The table compares the number of doses, vaccine volume, and vials required for each regimen. The variation in vials needed depends on clinic throughput and vial sharing practices for ID regimens, accounting for vial dead-space and daily discard of opened vials. We include the range in vials used, given no vial sharing (maximum of 1 bite patient per day) versus complete vial sharing (at least 5 patient presentations daily). We assume 1 mL vials, as per WHO prequalified rabies post-exposure vaccines [19,23]. STG = standard treatment guidelines in Tanzania.

Regimen	Description	Dose	Volume per course (mL)	Vials per course (range)
Off-label	One complete vial per IM dose over one month, widely used	3	3	3
UTRC	Two 0.1 mL ID injections per dose on days 0, 3, 7 and 28, recommended by WHO and STG, adopted in many facilities	4	0.8	1–4
1-week ID	Two 0.1 mL ID injections per dose, on day 0,3, and 7, recommended by WHO and for adoption in Tanzania	3	0.6	1–3

The use of abridged regimens could reduce vaccine doses and the cost per patient, making PEP both more affordable and accessible. Rabies vaccines cost between $6.60 and $20 per vial, with the total cost to patients further influenced by the indirect expenses of seeking PEP [9]. Bite patients in most African countries are usually required to pay the full cost of PEP, which is a barrier to seeking, starting, and completing PEP. Evidence from Tanzania, where patients typically pay for PEP, indicates that approximately 25 % of bite patients do not seek care, and over 15 % do not start PEP despite reaching health facilities [10]. The high cost of PEP not only discourages patients from seeking care but also places a substantial financial burden on the health system budget, limiting the capacity of healthcare facilities to maintain adequate stocks. As a result, PEP is accessible only in a limited set of urban public and private healthcare facilities, which are affected by frequent stockouts, forcing patients to seek PEP across multiple facilities or even in other districts [[10], [11], [12]].

Improving access to rabies vaccines, including decentralising their availability to multiple healthcare facilities within a district and offering them at no cost, improved patient outcomes when trialled in Tanzania previously [10,13]. In resource-limited settings, a further challenge lies in ensuring the judicious use of these costly biologicals. Unrestricted administration of rabies vaccines, especially to low-risk patients, can lead to shortages for high-risk individuals who urgently need PEP. Incorporating risk assessment through strategies like Integrated Bite Case Management (IBCM) may mitigate this challenge by minimising unnecessary vaccine use and ensuring priority access for high-risk cases [14]. Evaluating the economic and health impacts of policies associated with PEP access, including both WHO-recommended and locally used off-label regimens, is critical for identifying fit-for-purpose post-exposure vaccine provision that can be sustainably implemented in endemic regions where maintaining a consistent vaccine supply is challenging.

This study aims to examine the cost and effectiveness of current practice for human rabies vaccine provision in Tanzania [10] and compares them to the WHO-recommended one-week ID regimen [2], currently being considered for adoption in Tanzania and which is recommended for countries applying to Gavi, the Vaccine Alliance, for support to improve access to rabies post-exposure vaccines [15]. We employ a decision tree model using IBCM data from multiple regions in Tanzania to evaluate how different policies for PEP access could influence healthcare-seeking behaviours and resulting health and economic outcomes. The findings should inform policies in Tanzania and in other countries facing similar challenges, including how Gavi support can be leveraged for the most impact and for planning to improve post-exposure vaccine access.

## 2 Methods

### 2.1 Study overview

**Fig. 1:**
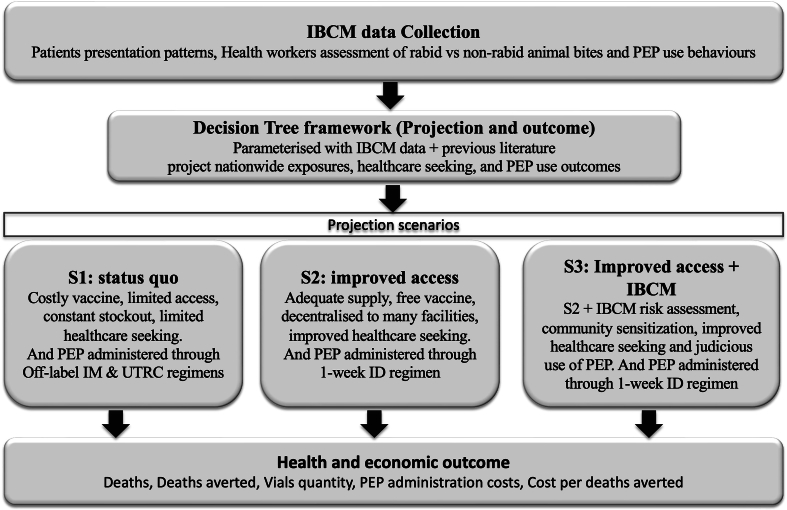
Framework for comparing the cost-effectiveness of PEP policies. The flowchart illustrates the components of the study, including the use of IBCM data from five regions to inform the decision tree model. The model projects nationwide outcomes across three scenarios: (S1) status quo with limited PEP access, (S2) improved PEP access and (S3) improved PEP access with IBCM implementation. Cost-effectiveness is analysed by comparing currently used regimens (S1) against the WHO-recommended 1-week ID regimen (S2-S3), and evaluating health and economic outcomes from the perspective of the health provider.

We analysed PEP provisioning practices, healthcare-seeking behaviours, and rabies risks using data from an IBCM programme implemented across four regions of Tanzania [14]. IBCM is a One Health approach to surveillance that links health and veterinary sectors to improve rabies case detection and patient management. The IBCM approach supports intersectoral collaboration through standardised patient risk assessments, investigation of biting animals and real-time reporting of exposures [14]. Informed by these data, we developed a decision tree model to estimate the health and economic outcomes of different PEP practices, evaluating deaths, costs, and cost-effectiveness per death averted across the Tanzanian mainland. The analysis was conducted from the health provider's perspective, comparing different policies for PEP provisioning at the facility level. Fig. 1 illustrates the study framework.

### 2.2 Data collection

***IBCM:*** We extracted IBCM data on bite patients seeking care between January 2019 and December 2024 across Mara, Mtwara, Lindi, and Morogoro regions. Information collected included bite patient demographics, location, biting animal details, exposure information, treatment given, and risk evaluation based on bite circumstances and animal history. Bites were categorised as high-risk for rabies if the biting animal was recorded by the health worker as (i) displaying at least one sign suggestive of rabies [16]; if (ii) it subsequently disappeared or died after the bite; if (iii) the bite was from a wild animal; or if (iv) the patient presented with signs of rabies [14].

***Costs:*** Using the microcosting valuation approach [17], we summarised the direct cost of providing PEP in public health facilities. We define each step of patient care, from registration on the first hospital presentation to consumables and vaccines for each dose and health worker time for vaccine administration. We included RIG costs for a supplementary scenario, with RIG administration for bites to the head and neck as these carry the highest rabies risk. We assumed that facility overheads (patient cards, vaccine storage, and other hospital infrastructure) were covered by consultation fees paid by patients. We excluded costs of wound treatment, as we aim to describe resources essential for PEP exclusively. Health workers' cost was defined as an opportunity cost of their time registering and administering PEP relative to the average net salary, using the most common health cadre responsible for immunisations. We calculated the health worker cost per patient based on the duration of consultations. We did not include costs of laboratory investigations of human rabies, as these are rarely done in Tanzania.

### 2.3 Analysis

***IBCM:*** Summary statistics from the IBCM data were calculated for patients presenting to facilities by district, region, demography, and risk category. The total number of bite patients presenting to facilities was used to calculate the annual incidence of care seeking using the projected population from the 2012 census [18]. Vaccine use estimates were based on IBCM records of doses administered. For IM regimens, we counted each dose as one vial, while for ID regimens, we accounted for vial sharing among patients attending healthcare facilities on the same day, as described previously [19]. RIG costs were assessed for individuals with severe bites or bites to the head and neck using dosages calculated from average weights and assuming equine RIG. We examined temporal patterns in PEP initiation rates by risk category.

***Costs:*** health provider costs were calculated from the IBCM data as:

$$\text{cost}=\left(N_{\text{consult}}\;x\;\mathrm{fee}\right)+N_{\mathrm{PEP}}\;x\;\left(\text{cons}+\text{time}\right)$$

where

*N*_*consult*_ *=* consulting patients.
*N*_*PEP*_ *=* patient visits where PEP received.
*cons =* consumables per dose (Table 1).
*time =* minutes per vaccination multiplied by the health worker cost per minute.
*fee =* initial consultation fee.

**Fig. 2:**
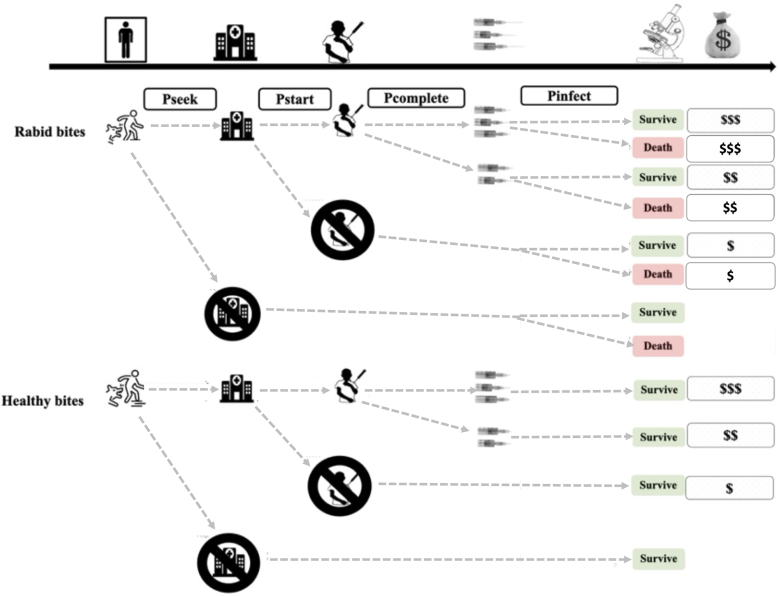
Decision tree of processes leading to health and economic outcomes. Victims of bites from healthy and rabid animals seek care with different probabilities (defined in Table 2), leading to different outcomes (vials used, deaths, costs, and cost per death averted)*.* Note that P_infect_ is conditional upon whether or not PEP has been provided and whether it was incomplete (Table 2).

***Decision tree model:*** We developed a model describing health-seeking behaviours that determine health and economic outcomes, as shown in Fig. 2 [20], described by the equations in the Supplementary materials. We assume bite victims of healthy and rabid animals seek care and start and complete PEP (defined as receiving three or more vaccine doses [10]), according to probabilities in Table 2, parameterised from the literature or the IBCM data. Specifically, risk assessments for IBCM allowed us to classify patients according to whether they were bitten by animals considered healthy versus probable rabid and therefore quantify variation in risk across the study populations. We compared alternative PEP policies under three scenarios: Scenario 1 (*S1*) representing the status quo; Scenario 2 (*S2*) representing improved PEP access, and Scenario 3 (*S3*) representing improved PEP access plus implementation of IBCM. Table 2, Table 3 detail the parameter values and costings used for each scenario, and we outline the rationale for our assumptions below.

**Table 2:** Scenario-specific probabilities used in the decision tree model. The three modelled scenarios (S1–3) are described in the methods. Parameters for rabid bite exposures are from previous work in Tanzania [10], [22]; those for healthy bites are based on the IBCM data. Probabilities were calibrated where appropriate to the IBCM bite patient presentation data, as described in the methods. For S2 and S3 an additional variant with RIG provided to patients with severe bite wounds or bites to the head and neck, is presented in the supplementary material.

Parameter description	Parameter	S1	S2	S3
Probability of seeking care dependent on the biting animal's status	P_**seek**,rabid_	0.75	0.87	0.9,
	P_**seek**,healthy_	0.25	0.375	0.375
Conditional upon seeking care, the probability of starting PEP is dependent on the biting animal's status.	P_**start**,rabid|seek_	0.847	0.907	0.907
	P_**start**,healthy|seek_	0.847	0.907	0.907
Conditional on having initiated PEP, the probability of completing the regimen is dependent on the biting animal's status.	P_**complete**,rabid|start_	0.54	0.70	0.85
	P_**complete**,healthy|start_	0.35	0.455	0.35
Conditional upon the level of PEP provided to a rabies exposure, the probability of developing rabies	P_**infect**|incomplete_	0.014	0.014	0.014
	P_**infect**|complete_	0.00	0.00	0.00
	P_**infect**|noPEP_	0.165	0.165	0.165

**Table 3:** Breakdown of resources and cost components for post-exposure vaccination. The breakdown includes total costs per patient for both complete and incomplete regimens. All costs were estimated during this study, with the unit cost sourced from the Tanzania Medical Store Department.

Variable	Description	Unit/Cost	Unit of measure	
*Consumables (cons)*				
Vaccine	Cost of vaccine vial	$10	Per vial	
RIG	Cost of equine RIG vial	$11	Per vial	
Syringe	Cost of syringes and needles	$0.06	Per Pc	
Gloves	Cost of pairs of clean gloves	$0.31	Per pair	

*Time cost (time = t*_*vax*_*t*_*hw*_*)*				
Vaccination time (*t*_*vax*_)	Minutes to administer the injection(s)	5	Per patient visit	
Health worker time (*t*_*hw*_)	Health worker salary	$0.06	Per minute	
	
*Consultation fee*				
*fee*	Registration fee charged on a first hospital visit, covering overhead	$1.3	Per initial visit	

*S1* represents the status quo, where PEP is typically only available from district hospitals, and patients pay for each vaccination they receive. Stockouts are common, although vaccines sometimes can be bought from private pharmacies for patients to bring to the hospital for administration. The probabilities that patients present to clinics and start PEP are parameterised from contact tracing data for rabies exposures [10], with calibration from the IBCM risk assessment data used to estimate these probabilities for those bitten by healthy animals, i.e., the remaining patient presentations (Table 2). We compared two post-exposure vaccination regimens used in Tanzania for *S1:* an off-label 3-dose IM regimen that is commonly used in Tanzania despite not being recommended [10] and the UTRC regimen [21], recommended in Tanzania's standard treatment guidelines (STG) and by the WHO (Table 1).

*S2* represents improved PEP supply, with more decentralised access and free provision. Specifically, vaccines are available from three additional health facilities within each district, as undertaken during a rabies elimination demonstration project in southeast Tanzania [13]. The hypothesised effect of this difference is increased clinic visitation rates for patients bitten by both rabid dogs (P_**seek**,rabid_) and healthy dogs (P_**seek**,healthy_), as shown in Table 2. Similarly, the probabilities of starting and completing PEP are higher in *S2* than in *S1*, as we expect patients to be more likely to initiate PEP at facilities where it is free and stockouts are less frequent. We consider only the 1-week abridged ID regimen for this scenario, which is recommended for countries applying to Gavi for rabies post-exposure vaccine support and has been recommended by Tanzania's National Immunisation Technical Advisory Group. We include RIG administration to patients with either severe bites or bites to the head and neck as an additional variant in the supplementary materials, as RIG has not been available in Tanzania until recently.

*S3* is the same as *S2*, except that it additionally involves training of health and veterinary workers in IBCM, which is also recommended for countries applying to Gavi. Under scenario *S3*, we hypothesise further improvements in health-seeking by rabies-exposed individuals, as IBCM investigations may reveal rabies-exposed individuals who did not seek care [24]. We assume a modest increase, with 20 % of those who did not seek care under *S2* seeking PEP under *S3* and improved PEP completion by exposures identified and counselled through IBCM, versus reduced PEP completion by bite patients assessed to be bitten by healthy animals which are not a rabies risk. For *S3* we also only considered the 1-week abridged ID regimen.

We used the IBCM data from the four regions to extrapolate high- and low-risk bite incidence and rabies deaths across the country, scaling by district-level population estimates from the national census [18]. Numbers of bite patient presentations for each district and year were drawn from a Poisson distribution, with lambda sampled from a uniform distribution spanning the minimum and maximum annual incidence observed across study districts and years. We then split patients into high- or low-risk groups using a binomial draw, where the probability of a bite being high-risk was randomly sampled uniformly from within the range observed for the proportion classified as high-risk across the study districts over the study period. Since these represent patients' presentations, we estimated total exposures (including those not seeking care) by dividing by baseline healthcare-seeking probabilities. Numbers of patients seeking, initiating and completing PEP according to risk were then projected from scenario-specific probabilities (Fig. 2, Table 2).

Within the decision tree, vaccine use was calculated according to the route of administration, and rates for completing (second and third) PEP doses for those bitten by healthy and rabid animals, and *P*_*co*__*mplete,healthy|start*_ and *P*_*co*__*mplete,rabies|start*_, respectively, as follows:

$$V_{\mathrm{IM}}=H\left(1+P_{\mathrm{co}mplete,healthy|start}+{P_{\mathrm{co}mplete,healthy|start}}^{2}\right)+E\left(1+P_{\mathrm{co}mplerete,rabies|start}+{P_{\mathrm{co}mplete,\;rabies|start}}^{2}\right)$$

where *V*_*IM*_ is the number of vials administered via the IM route, and *H* and *E* are the patients bitten by healthy and rabid animals. For ID regimens, we used the average number of vials per patient in low- and high-throughput facilities (*VPP*_*low*_ and *VPP*_*high*_ respectively) to pre-calculate rates of vial sharing according to previous estimates [19]. The quantity of vials (V_ID_) used was calculated as:

$$V_{\mathrm{ID}}={\mathrm{VPP}}_{\mathrm{low}}\left(H_{\mathrm{low}}+E_{\mathrm{low}}\right)+{\mathrm{VPP}}_{\mathrm{high}}\left(H_{\mathrm{high}}+E_{\mathrm{high}}\right)$$

where *H*_*high*_ and *E*_*high*_ are the numbers of patients bitten by healthy and rabid animals, respectively, starting PEP at high-throughput facilities, while *H*_*low*_ and *E*_*low*_ are patients in low-throughput facilities.

For each scenario, we ran 1000 iterations applied to the population of mainland Tanzania and summarised outcomes over a 5-year time horizon (2026–2030) according to projected population growth [18]. The iterations were performed to account for variability and uncertainty in the model parameters and to generate prediction intervals.

***Cost-effectiveness:*** The total cost of rabies prevention from the provider's perspective was calculated as the sum of the costs of patient registration and PEP administration (Table 3). We calculated the Incremental Cost-Effectiveness Ratio (ICER) for each scenario by comparing the additional cost and effectiveness relative to the status quo, *S1*, as:

$$\mathrm{ICER}=\frac{\mathrm{\Delta PEPcost}}{\mathrm{\Delta Effectiveness}}$$

where ΔPEPcost corresponds to the change in total PEP administration expenditure under each scenario, and ΔEffectiveness is the modelled change in the number of deaths averted. Cost-effectiveness was expressed as the cost per death averted under each scenario. Economic outcomes were discounted at 3 % per annum over a 5-year time horizon, with all costs converted to present values. Values were recorded in Tanzanian shillings (TZS) and converted to US dollars (USD) using a five-year average exchange rate of 1 USD per 2314 TZS [25].

***Sensitivity analyses:*** To address uncertainty in model parameters and determine the robustness of results, we conducted a one-way sensitivity analysis around scenario *S1*. Cost parameters were varied from 50 % to 200 % around baseline values. For rabies exposures, the probability of infection in the absence of PEP (P_**infect**|noPEP_) varied by 50 %, and given incomplete PEP (P_**infect**|incomplete_) was constrained between 0 % and 5 %. Healthcare parameters (probabilities of seeking care, starting and completing PEP for both rabid and healthy bites) were varied over plausible ranges from 0.2 to 1. Bite incidence and the proportion of high-risk bites were varied based on empirical extremes, with additional uncertainty bounds. Using 1000 Monte Carlo simulations per parameter, we assessed impacts on rabies deaths, deaths averted, vial use, PEP costs, and cost per death averted.

All analyses were conducted in R version 4.4.3 [26]. Data and code for reproducing the study are available at: https://github.com/Changalucha/PEP_cost_effectiveness. The CHEERS checklist [27] was followed to guide reporting.

### 2.4 Ethical approval

This research was approved by the Sokoine University of Agriculture Senate (SUA/PVM/D/2019/0005/09), the Ifakara Health Institute Review Board (IHI/IRB/No.16–2021), and Tanzania's National Institute for Medical Research (NIMR/HQ/R.8a/Vol.IX/3701).

## 3 Results

### 3.1 IBCM data

**Fig. 3:**
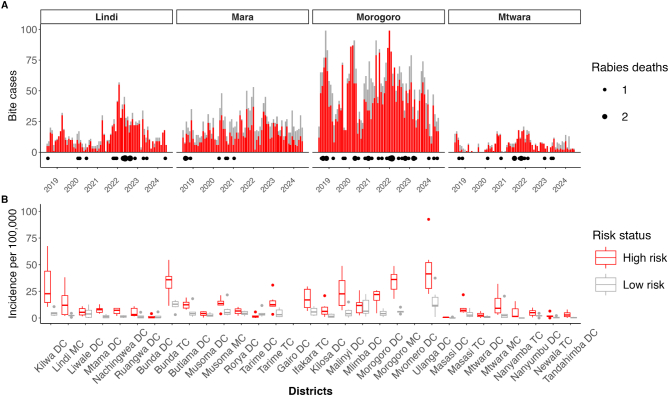
Bite patient presentations by exposure risk and human rabies mortality from 2019 to 2024. A) Monthly time series of bite patients and rabies deaths by region. B) Incidence of bite patients per 100,000 population across districts, categorised by risk (high risk = 5549 cases). Boxplots and whiskers show the interquartile range.

Between January 2019 and December 2024, we recorded 7423 bite patients seeking care for animal bites at health facilities providing rabies vaccines across the four regions (Mara, Mtwara, Lindi, and Morogoro). Total monthly presentations varied, ranging from just 46 (July 2022) to 233 (August 2024), with 74.8 % (5549/7423) of bite patients classified as high-risk (Fig. 3). Among the biting dogs classified as high-risk that were investigated by livestock field officers and from which samples were successfully recovered (*n* = 287), 91 % tested positive by rapid diagnostic test. Kilwa and Ulanga districts had consistently elevated incidences of high-risk bite presentations. The highest burden of bites was in children, with high-risk bites also concentrated in younger age groups (Table 4), including those to the head and neck. During this study period, 82 human rabies deaths were recorded. Morogoro (38 deaths) and Lindi (21 deaths) regions experienced higher rabies mortality compared to Mara (8 deaths) and Mtwara (15 deaths) regions.

**Table 4:** Demographic characteristics and incidence of bite patients and rabies deaths. Patients who presented across the four regions by year, age, and risk classification, including those who died of rabies.

Patient information	Features	All patients	High-risk patients	Rabies deaths
Total by year	2019	1104	805	14
	2020	1133	883	10
	2021	1103	773	12
	2022	1809	1410	26
	2023	1293	1038	16
	2024	981	640	4

Age	Mean	23.3	23.2	26.6
	Median	15	15	15
	SD	19.6	19.6	22.6

Age group	Under 15 years	3582	2697	40
	Above 15 years	3841	2852	42

Incidence (per 100,000)	2019	18.6	13.6	0.24
	2020	17.7	13.8	0.16
	2021	16.0	11.2	0.17
	2022	25.4	19.8	0.37
	2023	17.5	14.1	0.22
	2024	13.1	8.5	0.05

Substantial district variation in the annual incidence of bite presentations was observed, from 0.30 to 130 bites per 100,000 per year (0.30 to 93 high-risk bites per 100,000 per year, Fig. 3). Morogoro municipal and Ulanga districts both received the most bite patients and the most high-risk bite patients (993 high-risk/1106 total bites and 610 high-risk/820 total bites, respectively) compared to other districts. Some districts, despite having fewer total bite patients, had very high proportions of high-risk bite patients; for example, Tandahimba (89 %, 57/64) versus Kilwa (86.9 %, 538/619).

Vaccine administration practices varied across IBCM facilities, with 89 % (64/72) having experience using the UTRC ID regimen. However, the off-label IM regimen (Table 1) was used interchangeably with the UTRC at many of these facilities. The off-label regimen requires a larger vaccine volume compared to the UTRC and the 1-week ID regimen (Table 1). Over the six-year period, Abhayrab was the predominant vaccine brand routinely stocked and administered across IBCM facilities, though a small proportion of patients received Speeda.

Among patients who sought care, 6171 were recommended to start PEP, but only 5945 actually started PEP (of the 12,299 total doses recommended by health workers, 98 % (*n* = 12,045) were administered. Among those who started PEP, 5048 (91 %) were high-risk bite patients, whereas 164 high-risk bite patients that were recommended PEP were not recorded as being administered PEP. The rate of PEP initiation varied over the study period. Among high-risk patients, rates ranged from 95 % in 2019 to 97 % in 2024, with a notable dip to 77 % in 2021. Among low-risk patients, rates increased from 72 % in 2019 to 77 % in 2024, also showing variation in intermediate years. Of those who began PEP, only 37 % (*n* = 2182) completed at least three doses. From routine follow-up with the health officers in each district covered by the IBCM platform (23 districts) and investigation of human rabies deaths that were identified, we found no evidence of any patients developing rabies who had received timely post-exposure vaccination.

Of the administered PEP doses, 62 % (*n* = 7422/12,045) were given via the ID route, but its use decreased from 80 % in 2019 to between 42 % and 72 % from 2020 to 2024. On days when health facilities received bite patients, the median number attending per day was 1.6 (IQR: 1.3–2.0), with an overall average of 1.9 patients per day (95 % CI, 1.5–2.2, SD = 1.4). Approximately 29 % (21/73) of facilities treated at least two patients per day, with an average of 2.9 patients daily (SD = 0.46), highlighting the potential for vial sharing. For the IM regimen, where a full vaccine vial is used for a single patient, we determined the cost per dose to be $11.5 versus $6.6 per ID dose based on average patient attendance across facilities and accounting for discard of partially used vials. Our calculations illustrate how vial-sharing efficiency differs across facilities, resulting in ID costs ranging from $3.6 per dose (with optimal sharing at median facility attendance) to $11.5 per dose (with no sharing). From the IBCM data we estimate that 2.5 % of patients were bitten on the head or neck, and that these individuals had an average age of 13 years. Assuming RIG availability, these individuals would require an average of two RIG vials based on standard weight for age, resulting in a RIG cost of $22 per patient. If RIG were also given to patients with large or severe bites (9.3 % of bite patients, with an average age of 25 years), RIG requirements would increase to approximately 3 vials per patient, at a cost of $33 per patient.

### 3.2 Decision tree model

The model projects that, over a 5-year period from 2026 to 2030, approximately 59,000 exposures (95 % Prediction Interval (PI): 32,000-96,000) are expected to occur nationwide (Table 5). This equates to an average of 12,000 high-risk bites per year and does not change across the scenarios since there is no modelled change in dog vaccination that would reduce rabies transmission in dogs and therefore resulting rabies exposures in humans. The annual number of high-risk bites is projected to increase steadily, rising from 11,000 patients in 2026 to approximately 13,000 patients by 2030, which represents a 15 % increase over the five years.

**Table 5:** Projected health and economic outcomes under different PEP policies in Tanzania from 2026 to 2030. PI = 95 % prediction intervals.

Parameter	S1 Off-label & UTRC (95 % PI)	S2 ID 1-Week (95 % PI)	S3 ID 1-Week (95 % PI)
Patients starting PEP	58,000 (33,000–80,000)	77,000 (44,000–110,000)	79,000 (45,000–111,000)
Vials required	99,000 (56,000–139,000)	120,000 (68,000–171,000)	137,000 (78,000–191,000)
PEP cost in ‘000 US$	1040 (590–1450)	1280 (729–1821)	1470 (832–2035)
Deaths	3800 (2100–6200)	2300 (1200–3700)	2000 (1100−3200)
Deaths averted	6200 (3400–10,100)	7500 (4000–12,000)	7900 (4000–13,000)
Cost per death averted in US$	163 (135–211)	163 (129–225)	181 (143–243)

Under Scenario *S1* approximately 13,000 bite patients are projected to seek care every year, both exposures and those at low risk. Among these bite patients, 11,600 (95 % PI, 7000–16,000) are expected to initiate PEP, with an average of 5300 completing PEP. Around 800 [95 % PI, 400–1200] human rabies deaths are projected annually, cumulatively reaching 3800 by 2030 (Table 5). Current PEP use, which requires an annual average of 20,000 (95 % PI, 11,000–28,000) vaccine vials, is projected to avert approximately 1200 deaths each year under scenario *S1*. These vaccines would be administered at an estimated average cost of $206,000 annually. Consequently, the cost per death averted would average $163.

**Fig. 4:**
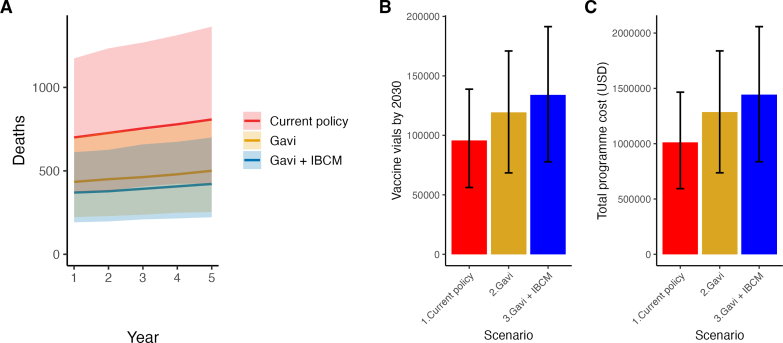
Projected health impact, vaccine demand, and programmatic costs of rabies PEP policies from 2026 to 2030. A) Annual rabies deaths under three policies over the 5 years. B) Cumulative vaccine demand in vials over a five-year horizon by access policy. C) Total costs associated with administering PEP over five years. Bars in panels B and C represent means, with vertical whiskers indicating 95 % prediction intervals.

**Fig. 5:**
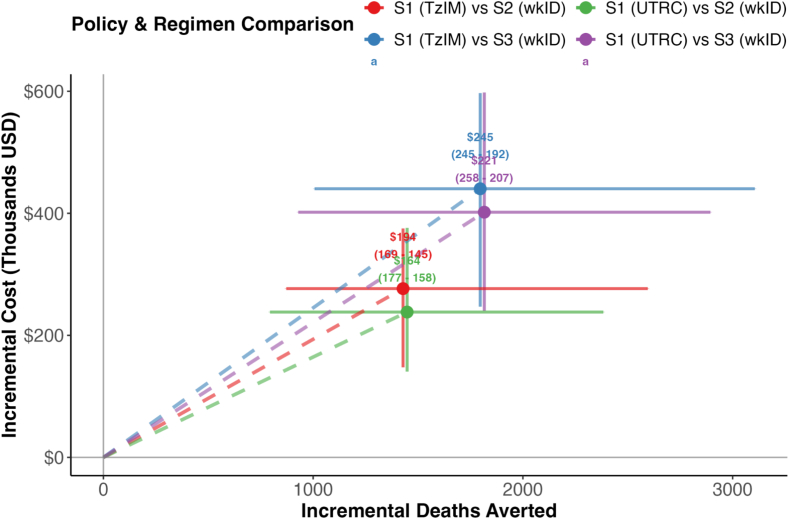
Cost-effectiveness plane comparing PEP access policies in mainland Tanzania. Incremental costs and deaths averted for improved PEP access policies (S2, S3) compared to the current practice (S1) over 2026–2030. ICER values (USD per death averted) are shown above each point. Solid lines show 95 % Prediction Intervals. Dashed lines represent ICER slopes from the origin. Both off-label (TzIM) and UTRC baseline regimens are compared against the 1-week ID (wkID) regimen.

Under improved PEP access scenario *S2*, health facilities are expected to receive an increase in bite patients compared to *S1*. On average, 16,000 patients would seek care annually, increasing from 15,000 (95 % PI: 7700-27,000) in 2026 to 18,000 (95 % PI: 9000–31,000) in 2030, resulting in a 33 % annual increase in patients starting PEP under *S2*. Of the additional 3700 annual patients starting PEP, 57 % are due to bites from healthy animals, representing a 1.6-fold rise in low-risk patients starting PEP. PEP completions are projected to average 9000 patients annually, driving vaccine requirements to around 24,000 (95 % PI: 14,000–34,600) vials per year. Rabies deaths are projected to be 430 (95 % PI, 220–700) in 2026, increasing to 490 (95 % PI, 260–790) by 2030, representing a 40 % reduction compared to *S1* (Fig. 4). The number of deaths averted is correspondingly expected to increase annually, from 1400 (95 % PI, 730–2300) to 1600 (95 % PI, 800–2600), at a cost of $163 per death averted (Fig. 5). If patients with either severe bites or bites to the head and neck were also to receive RIG, this would increase the cost of PEP by approximately 15–20 %, but without any detectable increase in lives saved, resulting in a cost per death averted of around $196 (Supplementary Table 1).

Under *S3*, the cumulative number of bite patients seeking care is projected to reach 85,000 by 2030 (Table 5). Annual projections indicate an average of 16,000 patients are expected to start PEP each year, with roughly 11,000 completing the full course. High-risk patients show improved completion rates in *S3* (99 % increase from *S1*) compared to the modest gain observed in *S2* (55 % increase from *S1*). *S3* is projected to require a cumulative total of 140,000 vials by the end of 2030 (a 14 % increase relative to *S2*), corresponding to an annual demand of 27,000 vials (95 % PI: 16,000–38,000 vials) administered at an annual cost of $290,000 (95 % PI: 166,000-408,000). Rabies deaths are projected to reduce over time, while the number of deaths averted is expected to rise from 1500 deaths averted in the first year to over 1700 deaths prevented by 2030, at an average cost per death averted of $180.

While the cost per death averted is lower for *S1*, most deaths are expected under this scenario. Our ICER analysis shows that both *S2* and *S3* offer greater health benefits at an additional cost (Fig. 5). At the national level, the ICERs ranged from $158 to $250 per death averted across *S2* and *S3*. The transition to *S2* was cost-effective at $158 (95 % PI: 163–151) per death averted, with a total of 1560 (95 % PI: 861–2500) deaths averted for an additional investment of $250,000 over the 5-year period. *S3* is expected to be the most effective with 1800 (95 % PI: 1000-3000) deaths averted nationally compared to S2 but requires higher investment ($430,000 over the 5 years compared to S1).

### 3.3 Sensitivity analysis

**Fig. 6:**
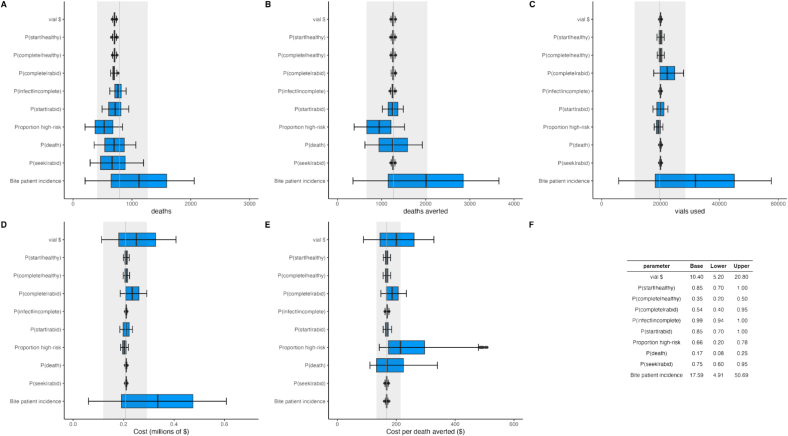
Sensitivity analysis of decision tree parameters influencing health and economic outcomes. One-way sensitivity analysis showing impacts of the ten most influential parameters on: A) rabies deaths, B) deaths averted, C) vaccine vials used, D) total costs, E) cost per death averted and F) the parameter range explored. Parameters are ordered by impact on deaths (see panel A). Boxplots show outcomes when each parameter is varied across its range (indicated in F) while others remain constant. Grey lines indicate baseline values with 95 % Prediction Intervals (shaded). Note that the baseline range of bite patient incidence largely defines these bounds. Cost parameters (vial, injection, healthcare worker, and registration costs) were excluded from display due to their minimal impact on outcomes compared to epidemiological and healthcare utilisation parameters.

The one-way sensitivity analysis identified bite incidence as the most influential parameter, strongly affecting numbers of deaths, deaths averted, vials used, and costs (Fig. 6A–D), but not cost-effectiveness, which was influenced most by vial cost (Fig. 6E). The next most influential parameters affecting rabies deaths were the probabilities of seeking care after a rabid bite (P_**Seek**,rabid_) and of dying from rabies in the absence of PEP (P_**infect**|no PEP_). In contrast, parameters related to bites from healthy animals had no impact on health outcomes and modest impacts on economic outcomes (P_**Seek**,healthy_ and P_**complete**,healthy|start_).

## 4 Discussion

### 4.1 Main findings

Our study predicts that expanding PEP access, particularly when integrated with IBCM, will reduce rabies deaths while achieving a favourable cost-effectiveness ratio. Modelled policies that expanded PEP availability resulted in higher care-seeking and treatment completion, with IBCM further enhancing vaccine targeting. Results from IBCM data revealed considerable variation in the incidence of bite patient presentations across years and between districts in Tanzania, with the majority representing likely rabies exposure as verified from investigations of biting dogs. These data informed model projections resulting in a wide prediction interval that consistently showed a high and increasing exposure burden driven by population growth. Between 2026 and 2030, about 59,000 exposures are projected, translating to over 16,000 bite patients every year, with 74 % classified as high-risk rabies exposures that could result in around two human rabies deaths per day under the status quo with inadequate PEP access. Policies that improve PEP access resulted in nearly a 41 % reduction in projected rabies deaths compared to the current practice. Incorporating IBCM further optimises outcomes by improving identification of patients at risk and prioritisation of high-risk patients, boosting PEP utilisation by 3 %, doubling completion rates among high-risk individuals, and reducing unnecessary administration among low-risk patients (who account for 57 % of additional PEP initiations under expanded access alone). This targeted approach achieves greater death prevention at a comparable cost per death averted. Under the current practice, limited PEP access is further strained by inefficient IM regimens that accelerate stock depletion, with stockouts recognized as a key reason for patients not starting PEP. Transitioning to the WHO-recommended 1-week ID regimen enhances programmatic efficiency, and when combined with IBCM, not only improves access but also maximises life-saving potential while preserving limited resources.

The projected vaccine requirements reveal resource implications for scaling access to PEP, with improved access policies increasing vial demand and PEP administration costs by 24–41 % from current practice. These cost increases must be weighed against substantial mortality reductions, and policymakers should prioritise life-saving impact given the favourable cost-effectiveness profile compared to other public health interventions. In this context, the goal of saving lives should also be supported by interventions that are fit for purpose: practical and deliverable within routine health systems. Integrating global guidelines with locally feasible practices allows efficient, equitable PEP scale-up. Moreover, Gavi investment would mean that under improved PEP access, governments do not pay the full costs of vaccine vials (see Supplementary Materials), incentivising policy implementation in the near term while reducing financial barriers that have historically limited PEP availability and building health system capacity for eventual transition to domestic financing.

Despite the overall improvement in the IBCM data in PEP initiation among high-risk patients compared to a previous study [10], both high-risk and low-risk patients experienced declines in 2021, suggesting a systemic factor. The discrepancy between PEP initiation and recommendation generally reflects issues such as vaccine stockouts or high costs to patients [9]. In addition, because patients lack information on which facilities actually stock vaccines, they often make unguided visits to different facility levels, both within and outside the district, which further disrupts efficient vaccine utilisation. Deviations from Tanzania's official treatment guidelines persist, as many facilities continue using off-label regimens [10]. The STG has endorsed both the UTRC and Essen regimens for over a decade, yet adoption remains limited due to inadequate training and supply chain constraints. The upcoming transition to the 7th edition of the STG, which replaces these regimens with the 1-week ID regimen as per WHO guidance [2], presents an opportunity to improve standardisation. However, successful implementation will require updated training for healthcare workers and careful resource planning to ensure proper adherence to protocols. This transition also demands upfront investment in vaccines, supply chains, and consumables, which could be supported by 10.13039/100001125Gavi financing to enable free provision and by the improved guidance developed by WHO [28].

IBCM data indicate that expanding the use of ID regimens could significantly reduce PEP costs. Although ID administration continues to be used, its uptake has fluctuated over time. Cost savings from ID administration depend on the vaccine vial volume and patient throughput at health facilities. Tanzania currently registers five rabies vaccine brands: Abhayrab, Rabivax-S, Vaxirab, Verorab, and Speeda, available in either 0.5 mL or 1 mL vials, with prices varying by manufacturer. Approximately 18 % of facilities treat more than two patients per day, making a 1-mL vial more economically efficient than 0.5 mL vials. However, since facilities have limited control over the vial sizes they receive, optimising the economic benefits of ID administration remains a challenge. Gavi support would address this constraint by procuring only WHO-prequalified vaccines in a standardised 1-mL vial size, using robust supply chains to ensure availability across facilities and facilitating uniform implementation of ID vaccination across healthcare facilities.

### 4.2 Strengths and limitations

This study has several limitations. First, the total number of bite patients seeking care reported through IBCM may be underestimated as a result of patients seeking care at non-IBCM facilities or at private pharmacies. Failing to account for all bite cases could impact the accuracy of the model results. We also used an overall bite incidence range derived from the study districts and applied this uniformly across all Tanzanian communities, which oversimplifies local variation influenced by human-dog relationships and population densities [29,30] and could greatly influence model predictions in a culturally diverse country like Tanzania. Nevertheless, a key strength of our study is the extensive multi-regional IBCM data and quantified parameters from prior research in Tanzania that captures variations= in PEP accessibility and utilisation patterns. By evaluating multiple PEP access policies, our study provides policymakers with a comprehensive framework for decision-making that is adaptable to local contexts. The implementation of the WHO/Gates rabies project in Tanzania (2010–2016) offered valuable insights into the practicality of improving access to free PEP while considering health system constraints in resource-limited settings [13].

A further limitation of our approach is that the model lacks certain parameters for accurately predicting health and economic outcomes. For example, the study did not account for the costs associated with implementing IBCM, particularly those associated with animal investigations (as well as the training required to establish IBCM), nor household costs associated with seeking PEP (such as travel and time lost). These additional costs would increase overall costs and should be quantified in future studies that take a more holistic approach to One Health. We also made assumptions about longer-term changes in health-seeking behaviour and access based on policy expectations which we are unable to validate. The magnitude of these potential changes is uncertain and should be monitored if policy recommendations are implemented. Our analysis focused solely on health facility expenses, overlooking the broader health system costs associated with supply chain management, vaccine wastage, training, and policy implementation, which could also substantially influence cost-effectiveness if accounted for. While issues like vaccine wastage and unpredictable demand have been considered in other work [19], these wider system costs could play a significant role in shaping PEP access and the disease burden, especially on a wider scale. For these reasons, it is important that Gavi-eligible countries such as Tanzania leverage Gavi investment to ensure that rabies vaccines benefit from strengthened vaccine supply chains and training and support for vaccine introduction and monitoring and evaluation.

### 4.3 Broader context

Our study shows that the cost per death averted is lower than estimates from countries like the Philippines, Haiti, and previous studies in Tanzania [22,31,32]. The favourable cost-effectiveness reflects the high proportion of rabies exposures among bite patients in Tanzania compared to other countries. The improved access scenarios are highly cost-effective, reinforcing the strong economic case for investing in rabies prevention through PEP. Additionally, the use of IBCM enhances case confirmation, ensures more accurate PEP prescriptions, targets high-risk bites, and supports community sensitisation for patients who might otherwise not seek care [14,31]. However, while improving PEP access is relatively inexpensive, these costs continue indefinitely without dog vaccination [33]. Dog vaccination programmes primarily reduce bite patient incidence by decreasing the overall rabies burden in the dog population. This also lowers the proportion of high-risk exposures. Integrating dog vaccination enables modelling of elimination timelines and better forecasting of how long PEP investment will be needed, which is critical for policymakers making informed decisions as the 2030 target to end dog-mediated human rabies approaches [34].

While all evaluated PEP regimens demonstrate epidemiological effectiveness in preventing deaths, their distinction lies in the efficiency of vaccine use. Recent efforts to optimise PEP have focused on reducing the number of doses and vaccine volume required, thereby enhancing patient compliance and cost-effectiveness [2]. This evolution is supported by evidence demonstrating comparable immunogenicity between IM and ID routes of administration and by studies showing that abbreviated regimens achieve protective levels of virus-neutralising antibodies [7,8]. Although WHO currently recommends both the one-week ID regimen and the four-dose IM regimen for PEP, the four-dose IM schedule was not explicitly modelled in this analysis. Direct comparison indicates that both regimens provide equivalent protection, but the IM regimen uses more vials per patient and as well as higher indirect costs per patient for the additional hospital visit required [35]. Consequently, the inclusion of the IM alternative would not alter the relative cost-effectiveness ranking of the improved access policy evaluated. There is scope for exploring other PEP vaccination regimens in future, with other ID regimens having potential for further efficiencies.

Expanding access to PEP may pose challenges for many rabies-endemic countries already burdened with multiple public health priorities. However, investments, such as Gavi's support for rabies vaccines, can potentially reduce the financial burden on both patients and governments (Supplementary Materials). To maximise the benefits of such investments, governments must redirect the alleviated resources toward dog vaccination on a national scale. Strengthening veterinary capacity through IBCM and dog vaccination would enhance efforts to control rabies and could improve the cost-effectiveness of preventing rabies and other zoonotic diseases.

As many Gavi-eligible countries transition out of support due to improved economic development, they will also be expected to fully fund their immunisation programmes, covering both routine vaccines and newly introduced ones, such as the rabies vaccine. This shift presents a future challenge to the financial sustainability of high-cost vaccines like rabies, especially in the absence of a coordinated procurement mechanism. Therefore, countries need to proactively assess the long-term financial implications and evaluate the potential benefits of leveraging national procurement agencies to achieve economies of scale and explore regional procurement coordination through mechanisms such as the East Africa Community, Southern Africa Development Community, or African Union. Such strategies could help maintain affordable vaccine pricing, similar to the current advantages secured through UNICEF's pooled procurement model.

## 5 Conclusion and recommendations

Given the favourable cost-effectiveness profile demonstrated across all scenarios, we recommend prioritising nationwide implementation of improved PEP access policies with IBCM integration. This approach not only maximises lives saved but also offers additional benefits not captured in our economic model, including strengthened health system capacity for bite case management, improved surveillance for rabies exposure, and enhanced community trust in preventive health services. While all modelled scenarios demonstrate life-saving potential compared to current practice, improved access strategies could reduce rabies mortality by nearly 50 %, translating into thousands of additional lives saved annually. The existing PEP practice in Tanzania falls short of meeting current demand, creating critical access gaps that perpetuate the rabies burden. IBCM integration further strengthens coordination between health, veterinary, and community-level prevention to deliver more equitable protection and greater overall mortality reduction. There remains an urgent need for dedicated policies to strengthen PEP access and leverage global initiatives such as Gavi to support sustainable investment. Adopting the WHO-recommended ID 1-week regimen alongside IBCM implementation could promote evidence-based practices for PEP use while supporting comprehensive health worker training.

The following are the supplementary data related to this article.

Fig. S1 Projected post-exposure vaccine demand and co-financing administration costs under different access policies. A) the number of human deaths per year, B) vaccine vials required by 2030 under the three policies and C) corresponding PEP costs by funding source (government vs. Gavi contribution). Fig. S1

Supplementary material 1 Image 1

## CRediT authorship contribution statement

**Joel Changalucha:** Writing – review & editing, Writing – original draft, Project administration, Methodology, Formal analysis, Data curation, Conceptualization. **Elaine Ferguson:** Data curation. **Martha M. Luka:** Data curation. **Kennedy Lushasi:** Investigation, Data curation. **Eleanor Rees:** Data curation. **Danni Anderson:** Formal analysis, Data curation. **Husna Hoffu:** Project administration. **Samweli Gwakisa:** Conceptualization. **Z. Mtema:** Software. **Maganga Sambo:** Conceptualization. **Kimera Sharadhuri:** Supervision. **Lwitiko Sikana:** Conceptualization. **Athumani Lupindu:** Supervision. **Jonathan Yoder:** Writing – review & editing, Supervision, Formal analysis, Data curation, Conceptualization. **Felix Lankester:** Writing – review & editing, Supervision, Funding acquisition, Formal analysis, Conceptualization. **Katie Hampson:** Writing – review & editing, Supervision, Project administration, Funding acquisition, Formal analysis, Data curation, Conceptualization.

## Funding

This work was funded by 10.13039/100010269Wellcome (207569/Z/17/Z and 224520/Z/21/Z), the UK Medical Research Council (MR/Z504919/1), and the US Department of Health and Human Services of the National Institutes of Health (R01AI141712).

## Declaration of competing interest

The authors declare that they have no known competing financial interests or personal relationships that could have appeared to influence the work reported in this paper.

## Data Availability

Data and code to reproduce the study are available at: https://github.com/Changalucha/PEP_cost_effectiveness.
