# Supplementary figures and images for: Comparative effectiveness and cost-effectiveness of policies for provisioning rabies post-exposure vaccines
Source: Vaccine. 2026 Feb 27;74:None. doi: 10.1016/j.vaccine.2025.128178 (PMC13201362; doi:10.1016/j.vaccine.2025.128178)

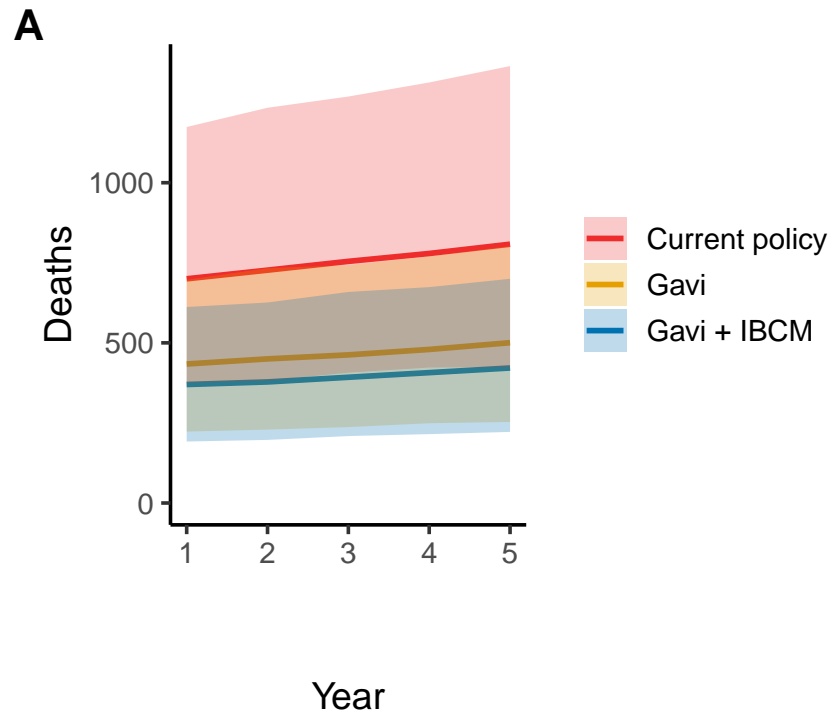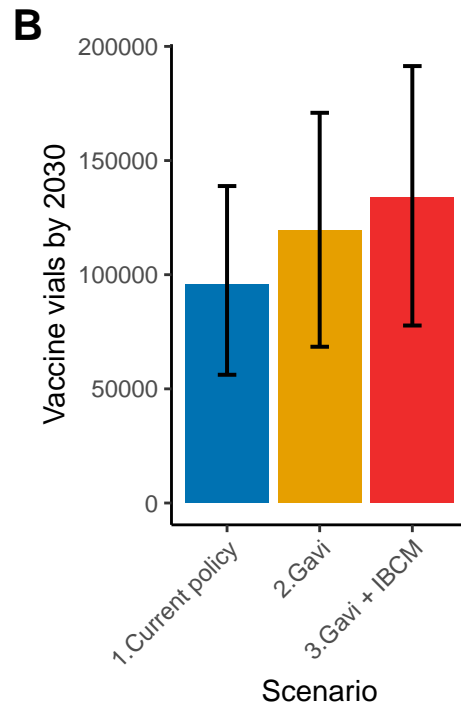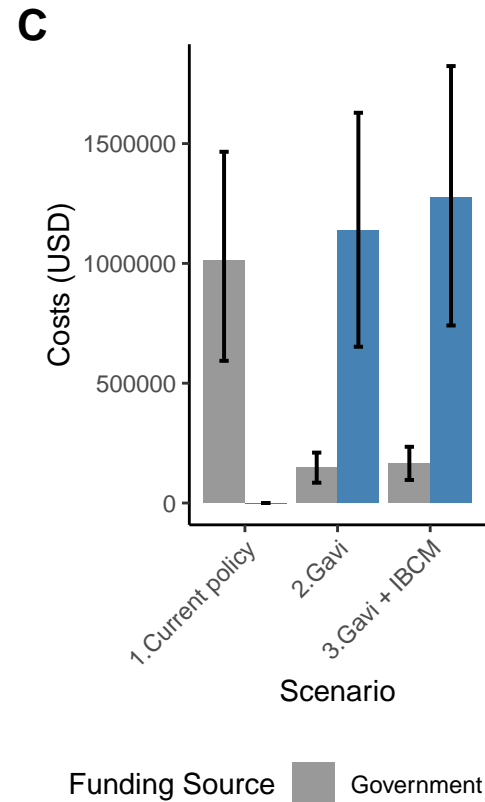

Supplement: Fig. S1 — Projected post-exposure vaccine demand and co-financing administration costs under different access policies. A) the number of human deaths per year, B) vaccine vials required by 2030 under the three policies and C) corresponding PEP costs by funding source (government vs. Gavi contribution). [file mmc1.pdf]
