# Supplementary material for: Comparative effectiveness and cost-effectiveness of policies for provisioning rabies post-exposure vaccines
Source: Vaccine. 2026 Feb 27;74:None. doi: 10.1016/j.vaccine.2025.128178 (PMC13201362; doi:10.1016/j.vaccine.2025.128178)
Supplement: Supplementary file 2 — Supplementary material 1 [file mmc2.docx]

## Supplementary Materials

Equations describing health-seeking behaviours that determine health and economic outcomes, as shown in Figure 2, are as follows:

Deaths_no_PEP_ = (Exposures * (1-P_seek,rabid_) * P_infect|noPEP_) +

(Exposures * P_seek,rabid_ * (1-P_start,rabid|seek_)) * P_infect|noPEP_)

Deaths_incomplete_PEP_ = Exposures * P_seek,rabid_ * (1-P_complete,rabid|start_) * P_infect|incomplete_

Deaths_complete_PEP_ = Exposures * P_seek,rabid_ * P_start,rabid|seek_ * P_complete,rabid|start_ * P_infect|complete_

Deaths_Total_ = Deaths_no_PEP_  + Deaths_incomplete_PEP_ + Deaths_complete_PEP_

Where: Exposures = High-risk bites/ P_seek,rabid_

PEP_start_ = (Exposures * P_seek,rabid_ * P_start,rabid|seek_) + (Healthy * P_seek,healthy_ * P_start,healthy|seek_)

PE_complete_ = (Exposures * P_seek,rabid_ * P_start,rabid|seek_ * P_complete,rabid|start_) +

(Healthy * P_seek,healthy_ * P_start,healthy|seek_ * P_complete,healthy|start_)

Within the stochastic decision tree model, outcomes were drawn from binomial distributions with denominators as shown above.

**Supplementary Table 1. Projected health and economic outcomes under improved PEP access policies in Tanzania from 2026 to 2030 with different levels of RIG provision.**  PI = 95% prediction intervals.

| **PARAMETER** | **S2, ID 1-Week, no RIG**  **(95% PI)** | **S2, ID 1-Week, RIG for head/ neck bites**  **(95% PI)** | **S2, ID 1-Week, RIG for severe bites and bites to head/ neck**  **(95% PI)** |
| --- | --- | --- | --- |
| PEP cost in '000 US$ including RIG | 1,280  (729 - 1,821) | 1,300  (769 - 1,862) | 1,490  (872 - 2,132) |
| Deaths | 2,300  (1,200 - 3,700) | 2,300  (1,200 - 3,700) | 2,300  (1,200 - 3,700) |
| Deaths averted | 7,500  (4,000 - 12,000) | 7,500  (4,000 - 12,000) | 7,500  (4,000 - 12,000) |
| Cost per death averted in US$ | 163  (129 - 225) | 168  (134 - 231) | 196  (152 - 265) |
